# Transcript-Level Biomarkers of Early Lung Carcinogenesis in Bronchial Lesions

**DOI:** 10.3390/cancers16122260

**Published:** 2024-06-18

**Authors:** Mikhail A. Pyatnitskiy, Ekaterina V. Poverennaya

**Affiliations:** 1 Institute of Biomedical Chemistry, Moscow 119121, Russia; k.poverennaya@gmail.com; 2 National Research University Higher School of Economics, Moscow 101000, Russia

**Keywords:** bronchial premalignant lesion, transcriptomics, biomarker, WGCNA, cilium

## Abstract

**Simple Summary:**

Premalignant lesions in the bronchial epithelium mark the early stages of squamous cell lung carcinoma and are difficult to detect using traditional methods. Therefore, there is a critical need to identify biomarkers that can aid in their detection and characterization. Here, we perform system-biology analysis of transcriptomic profiles of premalignant lesions with the aim of deriving novel biomarkers of bronchial lesions at the transcript level instead of the traditional gene-level approach. We found transcripts that are specifically associated with premalignant lesions along with several transcription factors that are likely potential regulators of the corresponding genes. The identified transcripts and transcription factors may serve as potential biomarkers or drug targets for prevention of squamous cell lung carcinoma. Furthermore, deeper understanding of the underlying biology may lead to the development of novel diagnostic tools and therapeutic strategies, impacting the broader research community engaged in lung cancer studies.

**Abstract:**

Premalignant lesions within the bronchial epithelium signify the initial phases of squamous cell lung carcinoma, posing challenges for detection via conventional methods. Instead of focusing solely on gene expression, in this study, we explore transcriptomic alterations linked to lesion progression, with an emphasis on protein-coding transcripts. We reanalyzed a publicly available RNA-Seq dataset on airway epithelial cells from 82 smokers with and without premalignant lesions. Transcript and gene abundance were quantified using kallisto, while differential expression and transcript usage analysis was performed utilizing sleuth and RATs packages. Functional characterization involved overrepresentation analysis via clusterProfiler, weighted coexpression network analysis (WGCNA), and network analysis via Enrichr-KG. We detected 5906 differentially expressed transcripts and 4626 genes, exhibiting significant enrichment within pathways associated with oxidative phosphorylation and mitochondrial function. Remarkably, transcript-level WGCNA revealed a single module correlated with dysplasia status, notably enriched in cilium-related biological processes. Notable hub transcripts included RABL2B (ENST00000395590), DNAH1 (ENST00000420323), EFHC1 (ENST00000635996), and VWA3A (ENST00000563389) along with transcription factors such as FOXJ1 and ZNF474 as potential regulators. Our findings underscore the value of transcript-level analysis in uncovering novel insights into premalignant bronchial lesion biology, including identification of potential biomarkers associated with early lung carcinogenesis.

## 1. Introduction

Premalignant lesions (PMLs) are early precursors of squamous cell lung carcinoma originating in the bronchial epithelium characterized by histological changes in the large airways, and are challenging to visualize with conventional bronchoscopy [1]. Autofluorescence bronchoscopy has been tailored for preinvasive lesion detection and has enhanced sensitivity in identifying such lesions [2]. However, a better understanding of the biology underlying transitions between different stages of bronchial PMLs may result in development of novel omics biomarkers, complementing existing diagnostic techniques [3,4].

Scientific interest in the early stages of squamous cell lung carcinoma has sparked the publication of several articles focusing on characterizing molecular alterations accompanying PMLs. A study published by Beane and colleagues [5] identified 280 differentially expressed genes in the airway field associated with premalignant lung lesions, revealing that several biological processes, including oxidative phosphorylation, electron transport chain, and mitochondrial protein transport, are notably upregulated. They also showed that bronchial brushes from normal-appearing areas of the mainstem bronchus can predict the presence of PMLs. A subsequent publication used gene expression to suggest that PMLs may be divided into four molecular subtypes (proliferative, inflammatory, secretory, and normal-like). Merrick et al. [6] investigated differences in gene expression profiles between persistent and regressive bronchial dysplasia, revealing 395 differentially expressed genes and 31 significantly altered pathways associated with cell-cycle control, proliferation, inflammation, and epithelial differentiation. Teixeira et al. [7] comprehensively profiled the genomic, transcriptomic, and epigenomic characteristics of carcinoma in situ lesions, revealing progression-specific methylation changes alongside a strong chromosomal instability signature.

While the aforementioned studies have primarily relied on gene expression analysis, an increasing volume of literature underscores the biomedical significance of protein isoforms, as these variants, originating from the same gene, can exhibit diverse biological functions and contribute differently to cellular processes [8,9,10,11], including oncogenesis [12,13]. In this study, in addition to the traditional gene-based analysis, we conducted a higher-resolution exploration of the transcriptomic profiles of both normal and PML samples, with a specific focus on protein-coding transcripts. We show that transcript-level analysis allows the detection of new biological processes and potential biomarkers of the earliest stages of lung cancer development, including possible targets for squamous cell carcinoma chemoprevention.

## 2. Materials and Methods

### 2.1. Transcript and Gene Quantification and Differential Expression Analyses

A total of 75 files in the SRA format were downloaded from the NCBI GEO resource, dataset identifier GSE79209 [5], and converted to the FASTQ format via the *fastq-dump* utility 2.11.3 (NCBI SRA toolkit) with the following parameters: skip-technical, readids, read-filter pass, dumpbase, split-3, clip. Quality trimming and adapter clipping were performed with *fastp* 0.23.4 [14], an ultrafast all-in-one tool for automatic cleaning of NGS data, including adapter removal, quality trimming, read filtering, etc. All settings for *fastp* were set to default values, which included automatic trimming of adapters and polyG tail, quality threshold Q20, and low-complexity threshold 30. After filtering, mean read length was equal to 98 bp, and on average, 93% of reads per sample passed all filters. For quality control, we used *fastQC 0.12.1* (https://www.bioinformatics.babraham.ac.uk/projects/fastqc/, accessed on 5 March 2024).

Transcript abundance in TPM units was estimated via *kallisto 0.50.1* [15], a popular lightweight tool for RNA quantification analysis. *Kallisto* uses an efficient pseudo-alignment approach that significantly reduces computational resources required to process large RNA-Seq datasets while maintaining high accuracy in abundance estimates. Furthermore, *kallisto* relies on known transcriptome annotation, making it a good choice for accurate RNA quantification in premalignant human samples, where the transcriptome is well studied and known isoforms are prevalent. We utilized *kallisto* with default settings except for a change in the number of bootstraps, which was set to 200. The index file for *kallisto* constructed from the Ensembl reference transcriptomes (version 108) was acquired from the website https://github.com/pachterlab/kallisto-transcriptome-indices (accessed on 11 March 2024). The abundance values for genes were obtained by summing corresponding TPM values for transcripts.

Differential transcript and gene expression was identified via the *sleuth* package [16], a tool for differential analysis of RNA-Seq data that was specifically designed to work with the bootstrap estimates from *kallisto* output. These bootstraps serve as proxies for technical replicates, allowing for accurate differential analysis of isoforms or genes. We used the Wald test implemented in *sleuth* with default settings, and a cutoff q-value was set at 0.05. Differential transcript usage (changes in the abundance ratios of transcript isoforms within a gene) was calculated using RATs 0.6.7 software [17], which is also able to take advantage of the bootstrapped quantifications from *kallisto* output. The following parameters were used: dprop_thresh = 0.1, use_sums = TRUE.

### 2.2. Functional Enrichment Analysis of Differentially Expressed Transcripts and Genes

All protein-coding transcripts and genes with significant differential expression (q-value < 0.05) were subjected to enrichment analysis using the *enrichWP* function from the R package clusterProfiler 4.10.1 [18], with maxGSSize parameter set to 200. Pathway content was downloaded from the WikiPathways resource [19]. Significantly enriched pathways (FDR less than 0.05) were visualized using the *dotplot* function.

### 2.3. Weighted Coexpression Network Analysis

All the protein-coding transcripts and genes were ranked by variance from large to small, and the log-transformed TPM values from the top 10% of features were selected as input for the weighted correlation network analysis using the R package WGCNA 1.72.5 [20]. This is a widely utilized algorithm for detection of highly correlated gene clusters that represent distinct biological processes. The *pickSoftThreshold()* function was used to screen the soft-threshold parameter, which ranged from 1 to 20. A soft threshold was selected to maximize the scale-free topology model fit. Automatic network construction and module detection was performed using *blockwiseModules()* function with the following parameters: pamRespectsDendro = TRUE, minModuleSize = 20, maxBlockSize = 4000, reassignThreshold = 0, mergeCutHeight = 0.25. The correlation between each gene/transcript expression values and the first principal component of each module (eigengene) provided a measure of the proximity between a gene/transcript and a specific module. Module eigengenes (first principal components) were correlated with available traits to identify modules that showed significant associations with clinical characteristics (COPD status, dysplasia status, maximum histology grade, packs per year, sex and smoking status). Pearson’s coefficient was used to calculate correlations, and the significance of coefficients not equal to zero was calculated using the *cor.test()* function.

### 2.4. Module of Interest Enrichment

Similar to the enrichment of differentially expressed transcripts and genes, all molecular features from the module of interest (protein-coding genes and transcripts) were subjected to enrichment analysis against Gene Ontology terms (biological processes and cellular components) using the *enrichGO* function of clusterProfiler [18]. Significantly enriched terms (FDR less than 0.05) were visualized using functions *treeplot* and *cnetplot*.

### 2.5. Analysis of Hub Transcripts

Module hubs were defined as transcripts that simultaneously satisfied two criteria: (1) detected by *sleuth* as significantly differentially expressed between normal and dysplasia classes (Wald test q-value less than 0.05), and (2) had absolute Pearson correlations between expression and first principal component (eigengene) of the “yellow” module greater than a threshold value of 0.93. Network visualization and enrichment analysis of the identified hub transcripts were performed using the Enrichr-KG web-server application [21]. All settings were set to default except for “Minimum links per gene” = 2. The following gene set libraries were used: Descartes_Cell_Types_and_Tissue_2021, WikiPathway_2021_Human, Gene Ontology (biological process), DisGeNet, and ARCHS4_TFs_Coexp.

## 3. Results

### 3.1. Identification of Differentially Expressed Transcripts and Genes

Transcriptomics data (GSE79209) obtained by Beane et al. [5] were downloaded from the NCBI GEO database (https://www.ncbi.nlm.nih.gov/geo/, accessed on 1 March 2024). To the best of the authors’ knowledge, GSE79209 is the only publicly available RNA-Seq dataset with raw data consisting of transcriptomic profiles of bronchial lesions. The initial dataset consisted of 82 samples and showed no significant differences in clinical traits, such as presence of chronic obstructive pulmonary diseases or reported smoking history among the subjects. Similar to the analysis performed by Beane et al. [5], brushes with the worst histology of metaplasia (*n* = 7) were excluded from subsequent processing. Reads were pseudo-aligned with the human transcriptome using the *kallisto* 0.50.1 software. This resulted in a total of 227,665 transcripts and 39,480 gene quantifications for each of the 75 samples.

As a first step, we conducted differential expression analysis, i.e., compared 25 samples with no evidence of PMLs (samples with no abnormal fluorescing areas or biopsies having normal or hyperplasia histology) against 50 samples with evidence of PMLs (biopsies having mild, moderate, or severe dysplasia) at the transcript and gene level.

Differentially expressed transcripts (DETs) were identified using the *sleuth* package [16]. A total of 84,625 transcripts passed the default initial filtering used by *sleuth* (at least five mapped reads to a transcript in at least 47% of the samples). According to the Wald test, there were 5906 DETs encoding for 4122 genes between normal and PML classes (q-value less than 0.05). Among the 5906 DETs discovered ~40% of transcripts belonged to the “protein_coding” class according to the Ensembl annotation, 37% transcripts were classified as “retained_intron,” and 10% transcripts were classified as “lncRNA”.

In order to compare between transcript-level and gene-level analysis, we also identified differentially expressed protein-coding genes (DEGs). According to the gene-level Wald test implemented in the *sleuth* package [16], there were 4626 DEGs between normal and PML classes (q-value less than 0.05).

**Figure 1:**
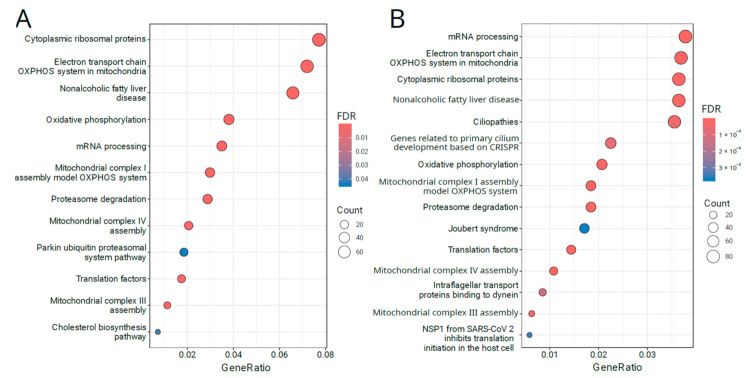
Functional enrichment analysis of the transcripts (**A**) and genes (**B**) differentially expressed between normal and PML samples. Gene sets were obtained from the WikiPathways resource [19], and the 15 most enriched gene sets are visualized. GeneRatio is the fraction of differentially expressed genes in the pathway of interest, contributing to the enrichment signal. False-discovery rate (FDR) is the adjusted *p*-value of the enrichment significance.

We performed functional enrichment analysis of the identified DETs and DEGs to identify biological pathways and processes associated with PML development. Results of the overrepresentation analysis using WikiPathways [19] as a source of pathway data are presented in Figure 1.

We report that pathways identified via DET-based enrichment analysis (1910 protein-coding genes) are consistent with the previous findings provided by Beane et al. [5]. There is significant enrichment of pathways related to cytoplasmic ribosomal proteins (FDR = 5.9 × 10^−55^), the electron transport chain OXPHOS system in mitochondria (FDR = 1.2 × 10^−38^), Nonalcoholic fatty liver disease (FDR = 1.8 × 10^−19^) and oxidative phosphorylation (FDR = 2.7 × 10^−18^). Full results of the DET functional enrichment analysis are available in Supplementary Table S1.

The DEG-based enrichment gave similar results to DET-based overrepresentation analysis, also identifying the abovementioned pathways like cytoplasmic ribosomal proteins (FDR = 2.4 × 10^−36^), electron transport chain OXPHOS system in mitochondria (FDR = 1.2 × 10^−28^), nonalcoholic fatty liver disease (FDR = 3.0 × 10^−10^) and oxidative phosphorylation (FDR = 8.1 × 10^−14^). Besides these, several cilium-related pathways were significantly enriched with DEGs, including ciliopathies (FDR = 4.1 × 10^−5^), genes related to primary cilium development based on CRISPR (FDR = 5.9 × 10^−5^), and Joubert syndrome (FDR = 3.8 × 10^−4^). Full results of the DEG functional enrichment analysis are available in Supplementary Table S2.

We then turned to the identification of differential transcript usage between normal and dysplasia classes. Using the transcript abundance quantified by *kallisto*, gene-level testing implemented in the RATs package identified two events of differential transcript usage: MRPS25 (mitochondrial ribosomal protein S25, FDR = 3.6 × 10^−5^) and COLCA1 (colorectal cancer-associated 1, FDR = 3.8 × 10^−22^). Although both genes are ubiquitously expressed in human tissue, we were unable to find evidence in the available literature connecting them to PML development.

### 3.2. Weighted Coexpression Network Analysis

To gain more insights into PML-related processes, we utilized weighted gene coexpression network analysis (WGCNA) [20] to identify coexpressed molecular features (transcripts, genes, proteins, etc.). In contrast to the enrichment analysis described in the previous section, WGCNA does not rely on the predefined gene sets: instead, correlated modules are identified de novo, thus offering a more holistic perspective on gene regulation and affected pathways.

Log-transformed transcript per million (TPM) values from the top 10% most variable protein-coding transcripts were used to derive the coexpression network (total 8343 transcripts). The soft-threshold parameter selected was 4 (Supplementary Figure S1). The same procedure with the abovementioned settings was performed for TPM values describing gene expression level (1870 most varying protein-coding genes). In this case, the soft-threshold parameter that maximized the scale-free topology model fit was 10 (Supplementary Figure S2).

A total of nine coexpression modules covering 6466 transcripts were identified by the WGCNA transcript-level analysis (Supplementary Table S3). There were 1877 transcripts classified by WGCNA as not being coexpressed and therefore assigned to the “grey” module. For the gene-level analysis, there were nine coexpression modules detected covering 1670 genes, while 200 genes were assigned to the “grey” module (Supplementary Table S4).

Each of the identified modules from gene- and transcript-level analysis was correlated with available sample metadata to reveal a subset of coexpressed molecular features (genes/transcripts assigned to module) significantly associated with clinical traits. The significance of correlation was estimated by *p*-value for the null hypothesis (correlation coefficient of zero).

**Figure 2:**
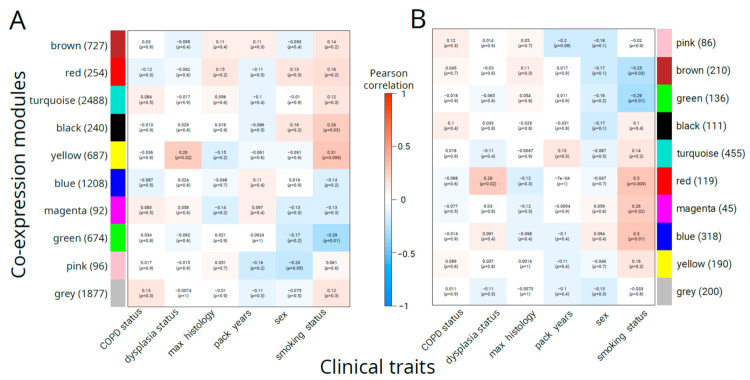
Correlation between WGCNA modules and clinical traits for transcript-level (**A**) and gene-level (**B**) analyses. Number of molecular features for each module is given in parentheses. Each cell contains Pearson’s correlation between module eigengene (first principal component) and the clinical trait. The *p*-value for the null hypothesis (correlation coefficient of zero) is given in parentheses.

For the transcript-level modules (Figure 2A), there was a single module (“yellow”) consisting of 687 transcripts that had a statistically significant correlation with dysplasia status (R = 0.26, *p* = 0.02), while the second-best correlated module (“brown”, 727 transcripts) had a non-significant correlation (R = −0.10 and *p* = 0.4). No modules were significantly associated with sex or COPD status. We found no modules that were significantly correlated with the worst histology. Several modules were found to be associated with smoking. The highest correlation (R = 0.31, *p* = 0.006) was observed for the “yellow” module, while the “green” module showed the second-best absolute correlation (R = −0.29, *p* = 0.01), followed by the “black” module (R = 0.26, *p* = 0.03). Overall, these results suggest that the “yellow” module contains coexpressed transcripts associated with the development of the precancerous disease state.

The results of WGCNA for gene expression values mostly coincided with transcript-level modules: a single module (“red”, 119 genes) was significantly correlated with dysplasia status (R = 0.28, *p* = 0.02), and five modules were correlated with smoking status: “red” (R = 0.3, *p* = 0.009), “blue” (R = 0.3, *p* = 0.01), “green” (R = −0.29, *p* = 0.01), “magenta” (R = 0.26, *p* = 0.02), and “brown” (R = −0.25, *p* = 0.03). There were no modules significantly associated with other traits.

### 3.3. Enrichment Analysis

We then turned to the biological interpretation of the “yellow” module for transcript-based analysis and “red” module for gene-based analysis, since both were found to be significantly correlated with dysplasia status.

**Figure 3:**
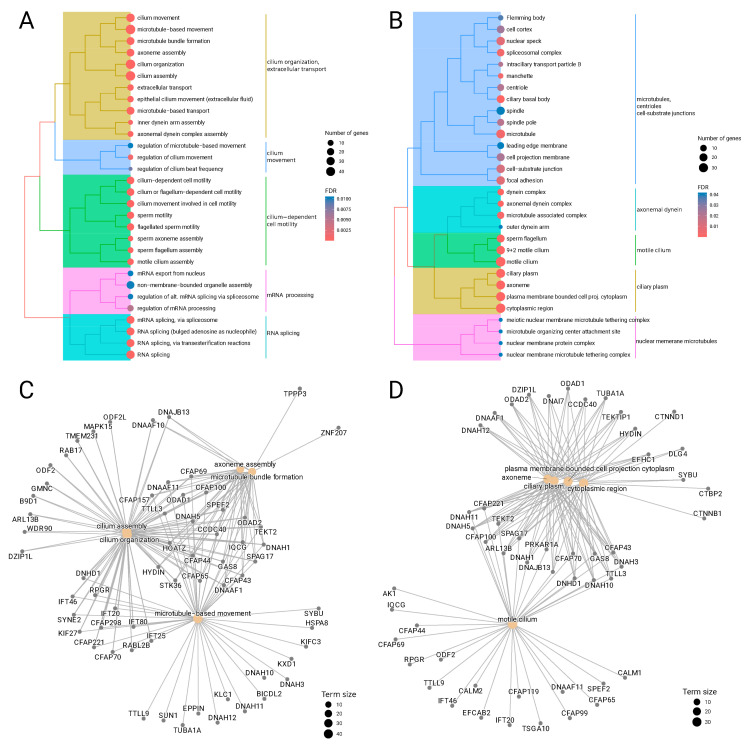
Functional enrichment analysis of transcripts belonging to the “yellow” module. Tree plots for significantly enriched Gene Ontology terms for biological processes (**A**) and cellular components (**B**). The circle size is proportional to the number of genes annotated as belonging to the term, and the circle color encodes the adjusted *p*-value of enrichment significance. Gene-concept networks for top five significantly enriched Gene Ontology terms for biological processes (**C**) and cellular components (**D**).

The transcripts of the “yellow” module were subjected to enrichment analysis against biological processes and cellular components described in Gene Ontology. Results of the analysis are visualized in Figure 3 in the form of tree plots and gene-concept networks. Tree-plot representation helps in obtaining a bird’s-eye view of the results by clustering related terms in the form of a dendrogram. Gene-concept network representation allows more detail by placing both enriched terms and corresponding genes on a graph. This enables the identification of genes that are involved in many processes simultaneously.

We report that the “yellow” module was significantly enriched in biological processes associated with cilium organization (GO:0044782, FDR = 4.2 × 10^−14^), cilium assembly (GO:0060271, FDR = 9.0 × 10^−15^), and cilium movement (GO:0003341, FDR = 1.5 × 10^−13^). Another cluster of enriched GO terms was also related to cilium activity and generally represents cilium-dependent cell motility (GO:0060285, FDR= 1.8 × 10^−8^). Finally, there was a cluster of significantly enriched terms not related to cilium and formed by biological processes related to mRNA splicing (GO:0000398, FDR = 1.3 × 10^−4^) and regulation (GO:0050684, FDR = 5.0 × 10^−3^). Expectedly, the analysis results for cellular components also demonstrated enrichment of the “yellow” cluster with cilium-related terms including axoneme (GO:0005930, FDR = 8.0 × 10^−13^), motile cilium (GO:0031514, FDR = 9.0 × 10^−13^), dynein complex (GO:0030286, FDR = 5.4 × 10^−5^) and others.

Gene-concept networks for the “yellow” module visualized several genes that are involved in multiple cilium-related biological processes and cellular components like DNAH5 (dynein axonemal heavy chain 5), CFAP221 (cilia- and flagella-associated protein 221), ODAD1 (outer dynein arm docking complex subunit 1), TTLL3 (tubulin tyrosine ligase-like 3), and HYDIN (axonemal central pair apparatus protein).

We found no significantly enriched biological processes for the gene-level “red” module. The top three processes were activation of GTPase activity (GO:0090630, FDR = 0.16), cilium movement (GO:0003341, FDR= 0.20), and axoneme assembly (GO:0035082, FDR = 0.21). There was a single marginally significant enriched cellular component ciliary basal body (GO:0036064, FDR = 0.04), while other cellular components were not significantly enriched, including axoneme (GO:0005930, FDR = 0.07) and ciliary plasm (GO:0097014, FDR = 0.07).

### 3.4. Network Analysis

We proceeded to identify the most significant transcripts that play a pivotal role within the “yellow” module, since the “red” module from gene-level analysis did not show significant enrichment. We defined hubs as transcripts that should satisfy two requirements. The first requirement was a high Pearson correlation of transcript expression with the “yellow” module “main direction” (first principal component), for which the threshold was selected as 0.93. The second requirement was significant differential expression between normal and dysplasia samples. There was a total of 16 transcripts that satisfied both requirements, listed in Supplementary Table S5.

We performed an in-depth analysis of identified hubs via the Enrichr-KG resource [21]. This web service combines gene set enrichment analysis with a knowledge graph of data representation and returns a network containing the top enriched terms from multiple libraries connected to the overlapping genes. All 16 hub transcripts of the “yellow” module were submitted to the Enrichr-KG. The following gene set libraries were selected: transcription factors from ARCHS4 coexpression [22], biological processes from Gene Ontology [23], cell types and tissue from Descartes [24], pathways from WikiPathways [19] and diseases from DisGeNet [25].

**Figure 4:**
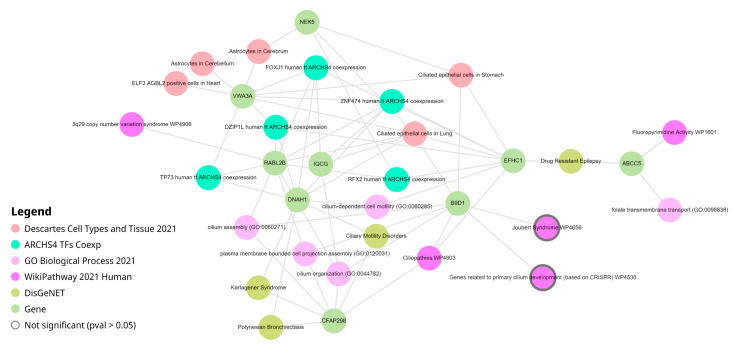
Network produced by Enrichr-KG, which links the top enriched terms with the hubs from the “yellow” module. Transcripts and genes with at least two connections to the neighboring nodes are visualized.

Results of the Enrichr-KG analysis are presented in Figure 4. The network consisted of 33 nodes connected with 63 edges. Consistent with the analysis of the whole “yellow” module described in the previous section, the network built for only hubs also indicated the involvement of various cilium-related bioprocesses (cilium organization, GO:0044872; cilium assembly, GO:0060271) and pathways (ciliopathies, WP4803). The most connected cell type was “ciliated epithelial cells in lung” (seven connections), followed by “ciliated epithelial cells in stomach” (four connections).

The most connected hub transcript was RABL2B (ENST00000395590, 10 connections). This gene encodes for GTPase, required for ciliation. Kanie et al. demonstrated that the RABL2B–GTPase complex, recruited by CEP19, plays a pivotal role in releasing pre-docked IFT-B complexes at the ciliary base, thereby initiating the entry of intraflagellar transport complexes into the cilium [26]. Three other hub transcripts, each with nine connections, were: DNAH1 (ENST00000420323, dynein axonemal heavy chain 1), EFHC1 (ENST00000635996, EF-hand domain-containing 1), and VWA3A (ENST00000563389, von Willebrand factor A domain-containing 3A). Of note, VWA3A has a total of five protein-coding transcripts, and three of them (ENST00000389398, ENST00000563389, ENST00000563755) passed the top 10% variability filter and were attributed to the “yellow” module.

Enrichment analysis identified several transcription factors that co-regulate selected hubs. Two of these transcription factors (FOXJ1 and ZNF474) regulate five hubs. While FOXJ1 is known to be the master regulator of motile ciliogenesis [27], the molecular function of ZNF474, a zinc finger protein, is rarely reported. Other identified transcription factors include DZIP1L, RFX2, and TP73. Transcription factor DZIP1L is located in the ciliary basal body and is known to be involved in cilium assembly and at the same time regulates Hedgehog signaling by interacting with GLI3 [28]. Transcription factor RFX2 coordinates multiple gene expression programs in the multi-ciliated epithelial cells, regulating cell movement, ciliogenesis, and cilia function [29]. Research is investigating the connection between RFX transcription factors and tumor formation and prognosis [30]. It was found that both protein and mRNA levels of ciliogenesis-associated markers FOXJ1 and P73 were significantly increased in patients with nasal polyps and associated with abnormal cilia architecture [31]. Autosomal-recessive deleterious variants in TP73 cause a mucociliary clearance disorder due to a defect in multi-ciliated cell differentiation [32].

## 4. Discussion

In the present study, we reanalyzed the dataset from Beane et al. [5] from two distinct perspectives. The first was the analysis of the differentially expressed transcripts as a complement to the traditional gene-based analysis. Transcript-level analysis provided a more detailed understanding of gene regulation and isoform-specific effects. The other perspective involved the application of weighted gene coexpression network analysis, a technique for the exploration of coexpressed genes and transcripts and capable of capturing subtle changes in expression patterns, which may not be detected by traditional overrepresentation analysis.

We were able to reproduce the main findings from Beane et al. [5], such as the identification of activated pathways related to oxidative phosphorylation and the electron transport chain. These pathways were identified by both gene- and transcript-level analyses. Of note, the connection between the PML-associated field of injury and processes linked to oxidative phosphorylation and the electron transport chain was experimentally validated through immunohistochemistry and bioenergetics studies.

Furthermore, transcript-level analysis coupled with WGCNA enabled us to discover additional biological processes related to the precancerous stages of squamous cell lung carcinoma. The main finding is the association between the development of premalignant bronchial lesions and dysregulation of cilium-related cell processes. While enrichment of differentially expressed genes also suggested involvement of cilia in PML development, it was transcript-level WGCNA that allowed to identify specific isoforms and confirm the results of differential enrichment.

Increasing evidence suggests the critical role of primary cilia in modulating various aspects of oncogenic signaling pathways, immunological responses, and inflammation [33]. It was found that primary cilia coordinate multiple signaling pathways, such as Hedgehog, TGFβ/BMP, G-protein-coupled receptors, WNT, and receptor tyrosine kinases to regulate developmental processes, tissue plasticity, and organ function [34]. Given the role of primary cilia in cell-cycle regulation, their involvement in tumorigenesis is plausible and supported by the dysregulated expression of cilium-related genes across various tumor types [35]. While limited data exist on airway cilia in lung cancer, histological changes from normal to dysplastic to cancerous tissue involve cilia loss, and marked downregulation of ciliated cell genes correlates with a more aggressive clinical phenotype in a subset of lung adenocarcinoma [36].

In our study, some cilium-related genes and transcripts were found to be associated with premalignant lesion development. Some of them are known to be linked with carcinogenesis. For example, tubulin glycine ligase TTLL3 knockdown decreased primary cilia and increased colon epithelial cell proliferation, promoting CRC development in mice and correlating with human CRC progression [37]. For other genes, such as VWA3A or EFHC1, we were unable to find significant confirmations in the literature. The discovery of a link between anticancer drug resistance and primary ciliary dynamics highlights the significance of primary cilia as a crucial target organelle for combating drug resistance in cancer treatment, thus emphasizing the need for research in this area [38].

Our analysis identified several transcripts that are deeply involved in the development of PML. Unlike DNA and proteins, transcripts allow for the immediate detection of cellular changes, making them promising biomarkers for early cancer detection and risk assessments [39]. However, ensuring the reliability and clinical utility of transcriptional biomarkers is a multistep and complicated process and faces many challenges. One of the main challenges in using RNA as a diagnostic analyte is its limited stability, which causes rapid fragmentation. Therefore, it is crucial to utilize robust and standardized protocols for RNA extraction that ensure high RNA yield and integrity. The candidate biomarkers should be retrospectively validated on an independent patient cohort, preferably with a bigger sample and using an orthogonal transcript detection method. For this purpose, RT-qPCR is commonly used, a well-established and sensitive technique for RNA quantification [40]. To avoid pitfalls with sample collection and handling, primer design and data analysis, it is advisable to follow the Minimum Information for Publication of Quantitative Real-Time PCR Experiments guidelines [41]. Prospective validation should be carried out through well-designed clinical trials; however, there are many issues associated with trial design and specimen and assay quality to be considered before the test is used in clinics [42].

There are several limitations of the presented study that potentially can limit the generalizability of the findings. One limitation is that bronchial brushes were obtained only from smokers, either current or former. Smoking is a significant risk factor for developing squamous cell lung carcinoma, as it creates an airway field of injury characterized by widespread molecular and cellular alterations throughout the bronchial epithelium [43,44]. This bias may overlook transcript expression patterns specific to non-smokers, limiting the study’s ability to capture the molecular mechanisms of PMLs in the broader population. Another limitation is the study’s cross-sectional design, which restricts the ability to infer causal relationships between transcriptomic changes and lung carcinogenesis development. While we found two bronchial lesion datasets with at least two time points per patient, one did not provide the raw data required for transcript-level analysis (GSE109743) and the other used microarray technology, which inherently has limited capacity for accurate isoform detection compared to RNA-Seq (GSE114489). The transcript-level analysis of longitudinal RNA-Seq data could help establish more subtle changes in isoform usage and expression compared with cross-sectional studies, thereby identifying early isoform-level biomarkers of PML progression, which can be a topic of subsequent research.

## 5. Conclusions

The presented transcript-level analysis provides insight into the isoforms underlying PML development, emphasizing the dysregulation of cilium-related processes. Identified transcripts and transcription factors may serve as potential biomarkers or drug targets for prevention of squamous cell lung carcinoma. However, further research into the role of cilium-related processes in lung tumorigenesis is warranted to develop novel therapeutic strategies.

## Data Availability

No new data were created or analyzed in this study.

## Supplementary Materials

The following supporting information can be downloaded at https://www.mdpi.com/article/10.3390/cancers16122260/s1. Figure S1: WGCNA diagnostics plot for transcript-level analysis; Figure S2: WGCNA diagnostics plot for gene-level analysis; Table S1: Enrichment analysis of differentially expressed transcripts; Table S2: Enrichment analysis of differentially expressed genes; Table S3: Description and WGCNA module assignment for top 10% most varying transcripts; Table S4: Description and WGCNA module assignment for top 10% most varying genes; Table S5: Hub transcripts of the “yellow” module.
