# Supplementary figures and images for: Transcript-Level Biomarkers of Early Lung Carcinogenesis in Bronchial Lesions
Source: Cancers (Basel). 2024 Jun 18;16(12):2260. doi: 10.3390/cancers16122260 (PMC11202239; doi:10.3390/cancers16122260)

### Scale independence

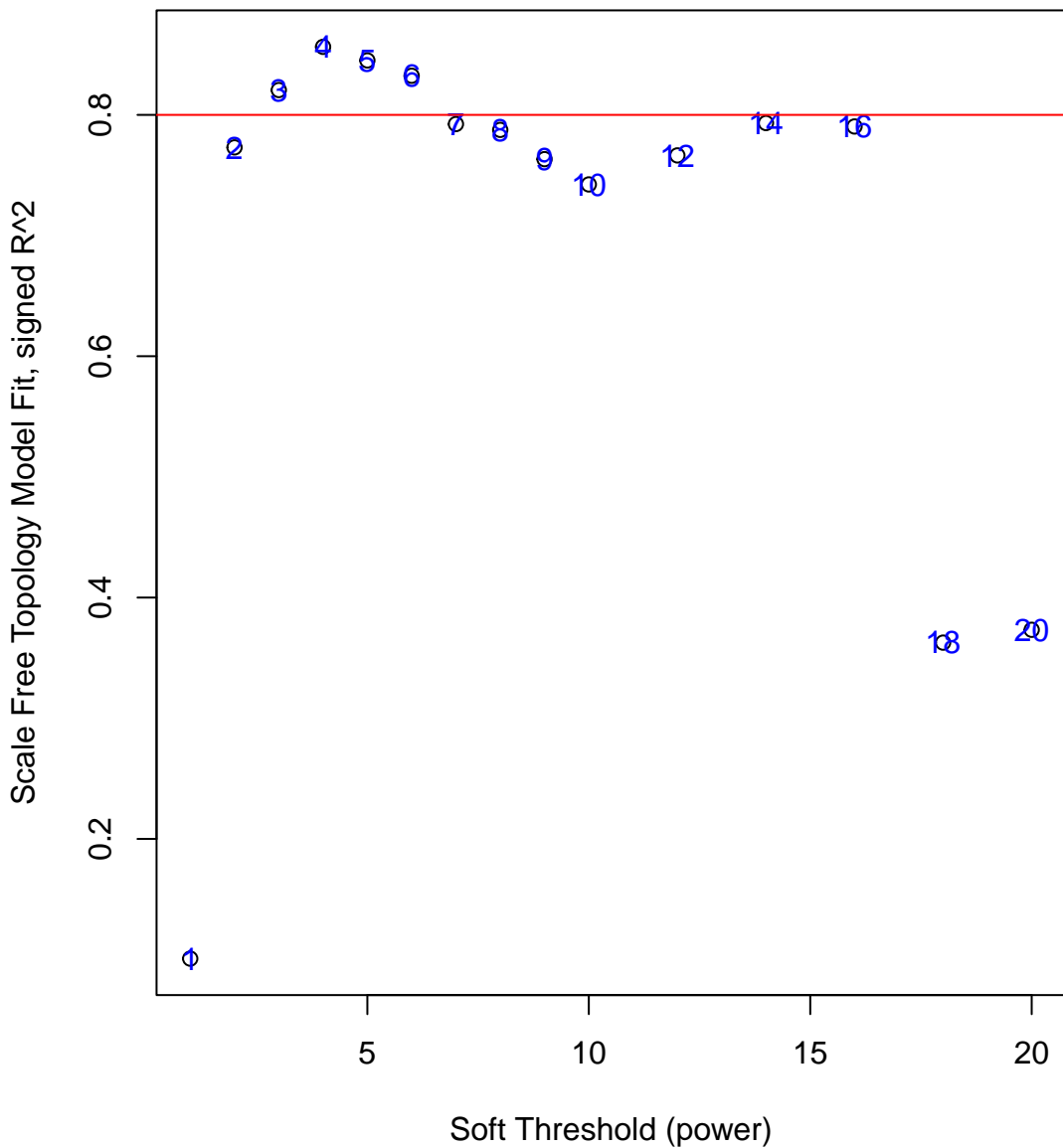

### Mean connectivity

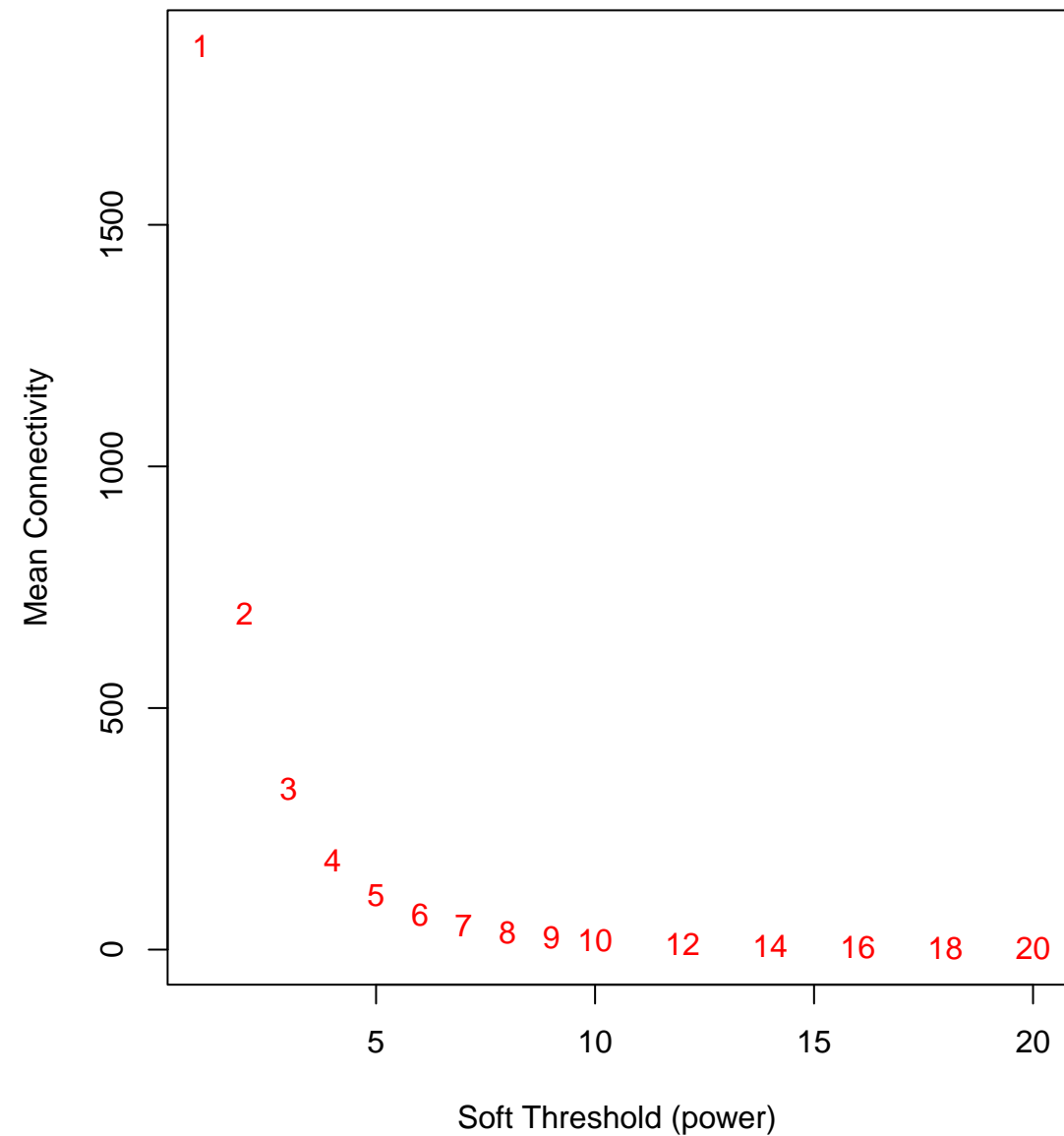

Supplement: Supplementary file 1 [file cancers-16-02260-s001.zip › Figure S1 WGCNA transcripts diagnostics.pdf]

### Scale independence

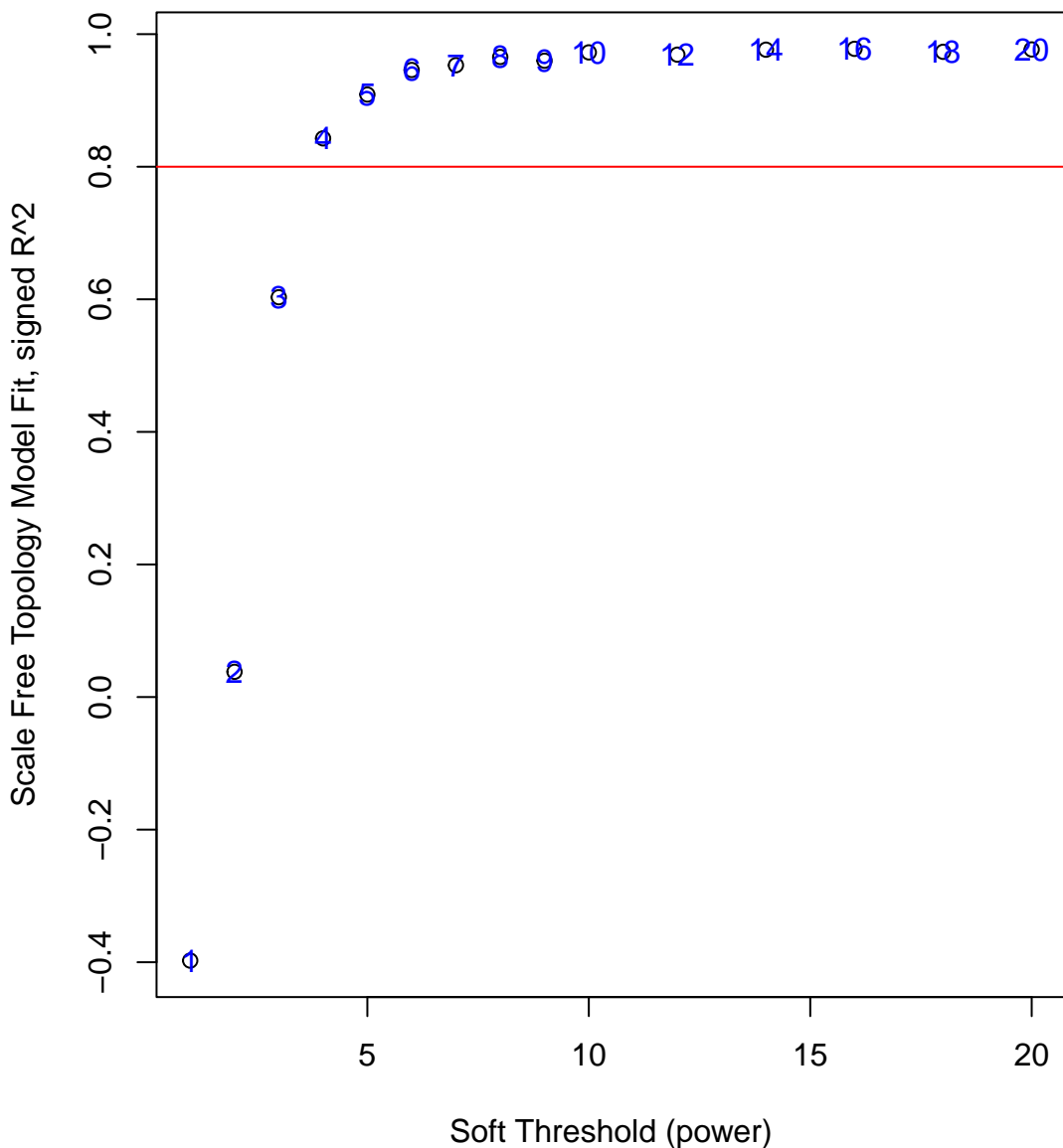

### Mean connectivity

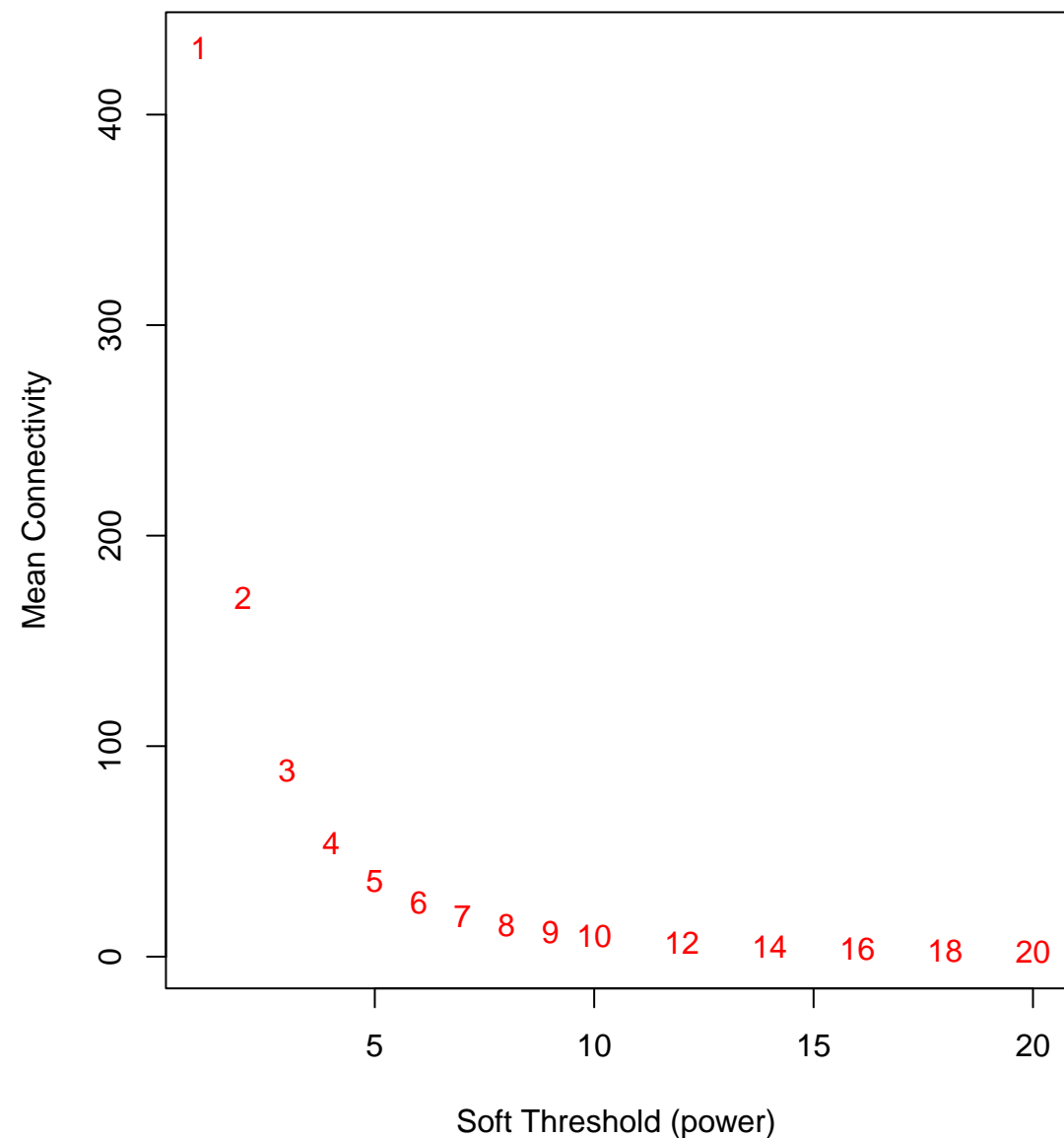

Supplement: Supplementary file 1 [file cancers-16-02260-s001.zip › Figure S2 WGCNA gene diagnostics.pdf]
